# Afebrile pneumonia (whooping cough) syndrome in infants at Hospital Universitario del Valle, Cali, 2001-2007

**Published:** 2012-06-30

**Authors:** Dolly Villegas, Connie Alejandra Echandía-Villegas, Carlos Armando Echandía

**Affiliations:** 1 Hospital Universitario del Valle, Cali, Colombia. E-mail: dvillegas@huv.gov.co; 2 Pontificia Universidad Javeriana, CaliColombia E mail: conicita5@hotmail.com; 3 Department of Pediatrics, School of Medicine, Faculty of Health, Universidad del Valle, Cali. Email: carlos.echandia@correounivalle.edu.co

**Keywords:** Pneumonia, whooping cough, bronchial spasm, pyloric stenosis, macrolides, *Chlamydia trachomatis*, Pneumonia

## Abstract

**Introduction:**

Afebrile pneumonia syndrome in infants, also called infant pneumonitis, pneumonia caused by atypical pathogens or whooping cough syndrome is a major cause of severe lower respiratory infection in young infants, both in developing countries and in developed countries.

**Objective:**

To describe children with afebrile pneumonia syndrome.

**Methods:**

Through a cross-sectional study, we reviewed the medical records of children diagnosed with afebrile pneumonia treated at Hospital Universitario del Valle, a reference center in southwestern Colombia, between June 2001 and December 2007. We obtained data on maternal age and origin, prenatal care, the childs birth, breastfeeding, vaccination status, symptoms, signs, diagnosis, treatment, and complications.

**Results:**

We evaluated 101 children with this entity, noting a stationary presentation: June-August and November- December. A total of 73% of the children were under 4 months of age; the most common symptoms were: cyanotic and spasmodic cough (100%), respiratory distress (70%), and unquantified fever (68%). The most common findings: rales (crackles) (50%), wheezing and expiratory stridor (37%); 66% were classified as mild and of the remaining 33%, half of them required attention in the intensive care unit. In all, there was clinical diagnosis of afebrile pneumonia syndrome in infants, but no etiologic diagnosis was made and despite this, 94% of the children received macrolides.

**Conclusions:**

These data support the hypothesis that most of these patients acquired the disease by airway, possibly caused by viral infection and did not require the indiscriminate use of macrolides.

## Abstract (es)

**Introducción:**

La neumonía afebril del lactante, llamada también neumonitis del lactante, neumonía por gérmenes atípicos o síndrome tosferinoso, es una causa importante de infección respiratoria baja grave en lactantes pequeños, tanto en países en vía de desarrollo como en países desarrollados.

**Objetivo:**

Describir los niños con neumonía afebril.

**Métodos:**

Por medio de un estudio de corte transversal, se revisaron las historias clínicas de niños con diagnóstico de neumonía afebril, atendidos en el Hospital Universitario del Valle, centro de referencia del suroccidente colombiano, entre junio de 2001 y diciembre de 2007. Se obtuvo información sobre la edad y origen materno, control prenatal, el nacimiento del menor, lactancia materna, estado de vacunación, síntomas, signos, diagnóstico, tratamiento y complicaciones.

**Resultados:**

Se evaluaron 101 niños con esta entidad, observando una presentación estacionaria: junio-agosto y noviembre-diciembre. El 73% de los niños eran menores de 4 meses, los síntomas más frecuentes fueron: tos espasmódica y cianosante (100%), dificultad respiratoria (70%) y fiebre no cuantificada (68%). Los hallazgos más frecuentes: estertores (50%), sibilancias y roncus espiratorias (37%). El 66% se clasificó como leve y del 33% restante, la mitad requirió unidad de cuidados intensivos. En todos se hizo el diagnóstico clínico de la neumonía afebril del lactante, pero no se realizó el diagnóstico etiológico y, a pesar de esto, el 94% de los niños recibió macrólidos.

**Conclusiones:**

Los datos presentes soportan la hipótesis que la mayoría de estos pacientes, adquirieron la enfermedad por vía aérea, posiblemente de etiología viral y no requerían el uso indiscriminado de antibióticos tipo macrólidos.

## Introduction

Acute respiratory infection (ARI) is the most common disease in humans. Infection of the lower airways, mainly pneumonia, in 2004 ranked third in the world as cause of death, considering all ages and the leading cause of mortality in children younger than five years of age^1^ ^-^ ^3^. In Colombia, pneumonia in 2005 ranked 16th as cause of disease burden, considering all ages and fourth place in children between 0 and 4 years of age^4^.

Afebril pneumonia in infants can cause severe respiratory distress, with atelectasis, apnea, hypoxic encephalopathy, seizures, and mortality, mainly in children under six months of age, premature infants, and infants whose mothers were very young^5^ ^,^ ^6^. It has also been found in studies of follow up, alterations in lung function (60%) with persistent wheezing bronchial obstruction (46%) and abnormalities in chest radiographs with bronchial thickening (15%)^5^.

A group of children with this syndrome, acquired through the birth canal of infected mothers or carriers of *Chlamydia trachomatis*, *Ureaplasma urealyticum*, and *Cytomegalovirus* ^7^. Another group, acquired by airborne respiratory syncytial virus, adenovirus, para-influenza, *Mycoplasma pneumoniae*, and *M. hominis*, without ruling out the possibility of *Bordetella pertussis* or *B. parapertussis* *^7^* ^,^ ^8^.

*Chlamydia trachomatis* is the bacteria most commonly found as a cause of afebrile pneumonia in infants^5^ ^,^ ^9^ ^-^ ^13^ and it is the most common sexually transmitted disease reported in the United States, with prevalence among pregnant women between 2 and 37%^5^ ^,^ ^9^.

Approximately 12 cases per month were received in the pediatric emergency department at Hospital Universitario del Valle in Cali, Colombia, for 4% prevalence. No tests were performed in these children to determine the causal agent and all were initiated in empirical treatment with macrolides to cover *C. trachomatis*, *U. urealyticum*, *Mycoplasma*, and *B. pertussis* and *B*. *parapertussis*.

Given the aforementioned, it was decided by this study to describe children with afebrile pneumonia syndrome (whooping cough), who attended a reference center, Hospital Universitario del Valle.

## Materials and Methods

Through a cross-sectional study, we reviewed the medical records of children admitted to the emergency department at Hospital Universitario del Valle, between June 2001 and December 2007, a reference center in southwestern Colombia, with the following inclusion criteria: young infants, with respiratory symptoms of rhinorrhea, dry cough, in entrances, spasmodic and cyanotic. We excluded patients with incomplete or lost medical histories.

### Sample size:

using the formula for descriptive studies, with a prevalence of 4%, a significance level of 95%, and a minimum expected difference of 5, we calculated a minimum of 60 patients^14^. 

### Measurements:

Information was collected on maternal age and origin, prenatal care, the place and way of the child's birth, gestational age, birth weight, breastfeeding, immunization status, neonatal hospitalization, sex, and age at admission, symptoms, signs, diagnosis, treatment, and complications.

### Plan of analysis:

the variables with missing data like maternal occupation, number of sexual partners, and home treatments administered to the child were removed. A description of the study population was performed. To describe continuous variables, measures of central tendency (mean and/or median as appropriate) were used, along with measures of dispersion: standard deviation (SD). Discrete variables were described with absolute and relative frequency distributions. Finally, we compared measurements between those who required or did not require intensive care unit by using relative frequencies, Chi^2 ^test, and for the means, the student t test.

### Ethical Aspects:

In keeping with the scientific, technical, and administrative standards for Health Research, Resolution No. 8430 of 1993 of the then Ministry of Health, this study was classified as without risks and evaluated and approved by the Human Ethics Committee of the Faculty of Health at Universidad del Valle and the Ethics Committee at Hospital Universitario del Valle.

## Results

**Figure 1.**
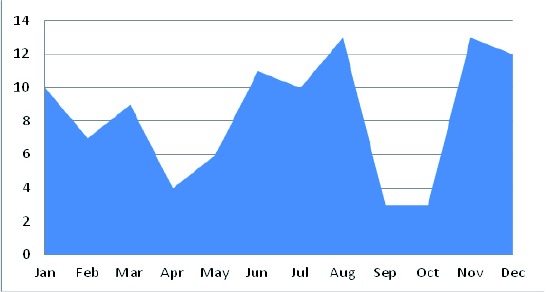
Distribution by afebrile pneumonia in infants HUV 2001-2007

In the 7-year period assessed, 101 children were admitted with a diagnosis of afebrile pneumonia in infants, noting that from 3 to 6 cases per year between 2001 and 2004, it increased to 17 to 34 cases per year after 2005. With a greater number of admissions during the months of June to August in the first half of the year and during November-December in the second half (Fig. 1).

The average age of mothers was 23 years, ranging between 14 and 42 years of age (SD 6 years), 14% of the mothers were minors, 54% from Cali, 33% from other municipalities in the department of Valle del Cauca, and 13% from other departments. Palmira (7%), Dagua (4%), and Sevilla were the most frequent municipalities of Valle del Cauca, and Puerto Tejada (3%), Santander (Cauca) (2%) and El Charco (Nariño) (2%) were the most frequent in other departments.

A total of 81% of mothers attended antenatal care, with an average of five controls, ranging between two and nine controls (SD 2). Eleven (16%) mothers reported vaginosis (eight treated) and five (8%) reported sexually transmitted disease during pregnancy (three treated): three with syphilis and one with papilloma virus.

Two of the children of mothers who reported syphilis during pregnancy were in the nursery at Hospital Universitario, one with Rh incompatibility and neonatal sepsis and the other with serological result of two dilutions and treated for 10 days.

Considering the children, 77% were born by vaginal delivery, 88% in a healthcare institution, the most common: Hospital Universitario del Valle, Hospital San Juan de Dios in Cali, and Hospital San Vicente de Paul in Palmira. Mean gestational age was of 38 weeks, ranging between 27 and 41 weeks (SD 3), 28% premature and average birth weight of 2,643 g, ranging between 720 and 4,000 g (SD 844), 7% weighing less than 1,500 g and 18% with low birth weight.

Neonatal hospitalization occurred in 28%, with an average stay of 18 days, ranging from two to 37 days (SD 14). The most common neonatal diagnoses were: hyaline membrane disease (8%), transient tachypnea (5%), and sepsis (4%). A total of 56% of the children received exclusive breastfeeding, three months on average, ranging between one and nine months (SD 2.6). A total of 58% were fully immunized, 29% had incomplete immunization, and 13% were unvaccinated.

The present illness had an average seven-day evolution, median of six days, ranging between one and 30 days (SD 6), with the most common symptoms: spasmodic and cyanotic cough (100%), respiratory distress (70%), unquantified fever (68%), and rhinorrhea(49%), as shown in Table 1.

**Table 1:**
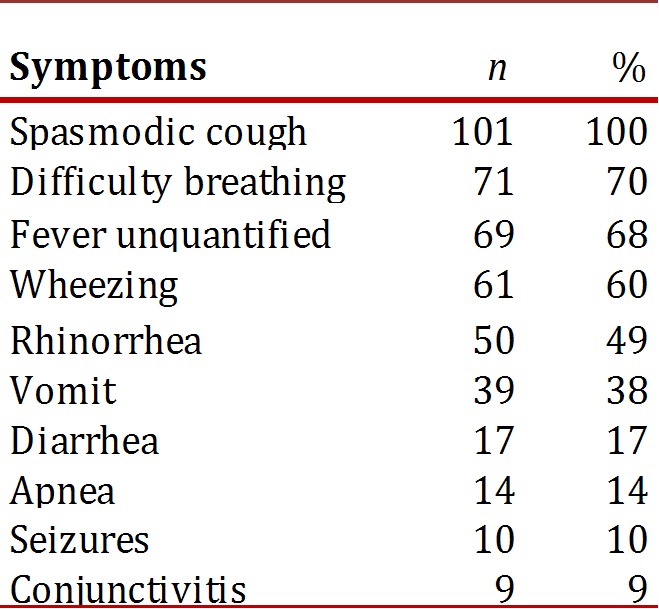
Most common symptoms of afebrile pneumonia ininfants, HUV 2001-2007

The average age at admission was six months, median of three months, ranging from one to 120 months (SD 13.4), younger than four months (73%) and 56% were male. Vital signs at admission were: average heart rate 149 beats per minute, ranging between 100 and 210 (SD 23); average respiratory rate 53 per minute, ranging between 10 and 99 (SD 15); average temperature 37.6 °C, ranging between 35 and 40 °C (DS 1.5); and average oxygen saturation 91%, ranging between 54 and 100% (SD 9).

During the initial physical examination, the most frequent findings were: rales (50%), wheezing and expiratory rhonchi (37%), and intercostal retractions (36%), as noted in Table 2.

**Table 2:**
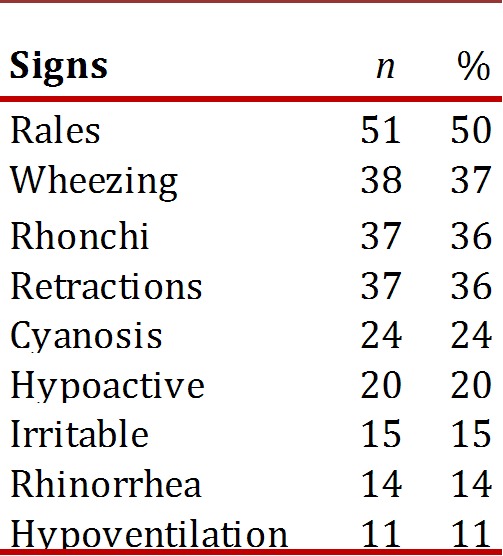
Most common signs of afebrile pneumonia in infants, HUV, 2001-2007

In the CBC, leukocytes averaged 18,488, ranging between 3,300 and 74,300 (SD 13,925), 39% of neutrophils, ranging between 11% and 90% (SD 18), 49% of lymphocytes, ranging between 8% and 84% (SD 18), 9.8 g/dl of hemoglobin, ranging between 2 and 16 g (SD 3), 496,842 platelets, ranging between 18,400 and 937,000 (SD 181,723), with leukemoid reaction (24%) and eosinophilia (6%).

In the chest X-ray report, the most common finding was pneumonia (37%), followed by interstitial infiltrates (26%), air trapping (20%), and atelectasis (10%); 10% of the reports describe the image of ''hairy heart.''

Only one medical story showed the result of immunofluorescence, in a nasopharyngeal swab sample, which was negative. No results were found of culture or serum antibody titers in the other children.

The most frequent treatment received by these children was macrolides (94%): half with erythromycin, 36% with clarithromycin and 8% with azithromycin. Oxygen was required for 72% of the children, mainly through cephalic chamber, followed by nasal cannula and 12% required intubation. B2 stimulant bronchodilators were received by 75% (two thirds via inhalation), ipratropium bromide by 26% (two thirds micro-nebulized) and 26% received steroids (most intravenous methylprednisolone); 65% received respiratory therapy management Table 3).

**Table 3:**
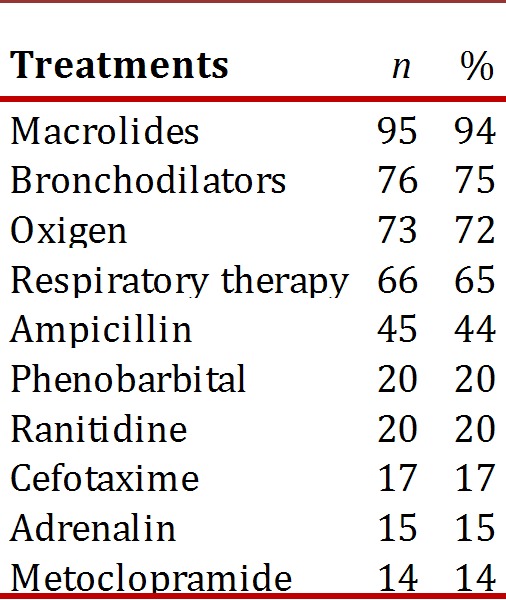
Most common treatments for afebrile pneumonia ininfants, HUV, 2001-2007

Upon admission, 66% of the children were classified as mild, 16% were discharged, and 50% were sent to level II care. The remaining 33% was rated moderate to severe, half required intensive care unit (17%) and the other half, hospitalization in the general ward. The average stay was four days, median one day, ranging between one and 28 days (SD 6.4).

## Discussion

One of the findings in this group of children was the stationary presentation of afebrile pneumonia, with two peaks of higher admissions: from June to August and from November to December, which is in favor of most cases; the etiology was viral because of its higher air transmission at certain times of the year and did not happen by germs in the vaginal canal during childbirth.

This finding is consistent with those reported by Franco *et al*., in 10 children under two years of age admitted to the emergency department of a clinic in Bogota, with acute respiratory infection, which by immunofluorescence in mucus nasopharyngeal samples taken by swabs determined that the viral etiology was predominant: three children with Respiratory Syncytial, three with Influenza, and one with Parainfluenza, also prevailing during April-May and October-November^15^.

Another result was that the clinical diagnosis was made according to data reported in the literature: predominantly children of few months of life, 73% were younger than four months of age, with a median of three months. The presence of cough in access, described as staccato, pertussis or whooping on all children and difficulty breathing, given by bronchospasm (66%), pneumonia (37%), and atelectasis (10%) with the corresponding respiratory therapy management with oxygen and bronchodilators^5^ ^,^ ^7^ ^,^ ^9^ ^,^ ^15^ ^,^ ^16^.

A retrospective study of 8 patients with afebrile pneumonia in infants (whooping cough syndrome), diagnosed by direct immunofluorescence, showed a median age of three months, ranging from 10 days to 19 months, all with respiratory distress, half with spasmodic cough, emetic; with the radiological findings of diffuse reticular infiltrates, air trapping, and excellent response to oral erythromycin^16^.

The percentage of children referred regarding unquantified fever during the current illness (68%) was not consistent with the literature. Children with this entity are described as afebrile and without toxic appearance, being caused by viruses and/or atypical bacteria such as *Mycoplasma*, *C. trachomatis* and *C. pneumoniae* ^15^ ^-^ ^17^. Part of the explanation is that 37% of the children had pneumonia by bacterial infection.

In a report of diagnosis and treatment of childhood pneumonia, Velasco *et al*.,^7^ described that in the etiology of afebrile pneumonia in infants, there is frequent co-infection between bacteria and viruses, between viruses and viruses, or between bacteria and bacteria. Sant´Anna *et al*., reported, as a main cause of bronchiolitis, the respiratory syncytial virus, followed by *B. pertussis*, *C. trachomatis, M. pneumoniae* and *Moraxella catarrahalis* with the concomitant presence of respiratory syncytial virus^18^. It wasn´t predominant the history of very young mothers with low socioeconomic conditions, without prenatal care, with a history of vaginal discharge, birth by vaginal delivery, lack of breastfeeding, or the presence of neonatal conjunctivitis, when this syndrome is produced by *C. trachomatis* ^5^ ^,^ ^7^ ^,^ ^9^ ^,^ ^15^ ^16^.

Only nine children had conjunctivitis, compared with the report of 50% chance of developing conjunctivitis when the germ that causes the syndrome is *C. trachomatis* ^9^. Only one mother of these nine children was a minor, eight attended prenatal care, none reported vaginosis during pregnancy, four children were born by caesarean section and five were admitted between June and July.

Of 11 mothers who reported vaginosis during pregnancy, eight were treated, only two were minors, 10 attended prenatal care, all with birth by vaginal delivery and none of the children developed conjunctivitis.

As for the etiological diagnosis in this group of children, only one revealed the result of immunofluorescence in nasopharyngeal secretion, which was negative. Despite this, 94% of the children received macrolide management. Some authors recommended early identification of the germ in children with this disease, which allows programming management at the hospital or at home, taking preventive measures of isolation and avoiding the indiscriminate use of antibiotics^15^.

In the report of 10 children with severe respiratory infection in Bogota, all wheezing and without fever, one in whom pneumonia by *C. trachomatis* was suspected, because of the presence of conjunctivitis and pneumonitis, Parainfluenza was isolated and macrolides were not used ^15^. Other authors recommend that the combination of clinical and radiological parameters, positive for this entity, allows initiating empirical therapy with macrolides, without the etiologic agent^5^ ^,^ ^19^ ^,^ ^20^ ^.^

There is evident association between the use of oral erythromycin in children under one month of life and the development of pyloric stenosis, along with the ignorance of this risk after treatment with other macrolides, azithromycin or clarithromycin type, and reports of resistance to macrolides between 30% to 60%^6^ ^,^ ^7^ ^,^ ^9^ ^21^ ^,^ ^22^.

When the etiologic agent is *C. trachomatis*, in addition to the initial isolation because of its highly contagious characteristic featuring up to 70 to 100% of diseases in health care staff or direct contacts, it is recommended to investigate other sexually transmitted infections on the child and assess the mother and her sexual partner^5^ ^,^ ^9^.

These data support the hypothesis that most of the patients reviewed, acquired the disease by airway, possibly caused by viral infection and did not require antibiotics such as macrolides.

According to data reported in the literature, we found cases of severe respiratory distress requiring intensive care unit for 17% and seizures in 10%. Finding in them greater proportion of atelectasis (OR 21, 95% CI 4.47 - 101.6), apnea (OR 11.5, 95% CI 3.26 - 40.8), retractions (OR 5.6, 95% CI 1.80 - 17.7), leukemoid reaction (OR 4, 95% CI 1.28 - 12.2), pneumonia (OR 3.7, 95% CI 1.05 - 13), and bronchospasm (OR 3.6, 95% CI 1.03 - 16.8) as compared to the group of children who did not require intensive care. These features become markers of severity, implying more care in the pediatric emergency department to the child with them.

The weaknesses of this study are that it was descriptive, cross-sectional, limited to a population that attending a referral center, and the information was obtained from the medical records of the institution, which have shown missing data. The strength of the study is the number of patients evaluated with this entity.

## Conclusions

These data support the hypothesis that most of these patients acquired the disease by airway, possibly caused by viral infection and did not require the use of antibiotics; despite this, 94% of the children received macrolides. This descriptive study provides insight into the epidemiology of the disease and the need to modify the local guides.

It is recommended to conduct a prospective study, taking nasopharyngeal swab of each patient admitted with this diagnosis to determine the etiologic agent and define its appropriate management.

## References

[1] Lozano JM (2006). Epidemiología de las enfermedades respiratorias en la niñez.En.

[2] World Health Organization (2008). Global burden of disease:.

[3] Black RE, Morris SS, Bryce J (2003). Where and why are 10 million children dying every year. Lancet.

[4] Rodríguez J (2008). Carga de la enfermedad en Colombia..

[5] Escamilla JM, Reyes MA, Aristizábal G, Leal FJ (2006). Síndrome: neumonía afebril del lactante. Neumología Pediátrica.Infección, alergia y enfermedad respiratoria en el niño.

[6] Centers for Disease Control and Prevention (2005). Outbreaks of Pertussis associated with hospitals - Kentucky, Pennsylvania, and Oregon, 2003. MMWR.

[7] Velasco MV, Pérez R, León C, Villafruela C (2005). Diagnóstico y tratamiento de las neumonías infantiles adquiridas en la comunidad. BSCP Can Ped.

[8] Centers for Disease Control and Prevention (2006). Pertussis outbreaks in an Amish Community - Kent County, Delaware. MMWR.

[9] Pickering LK, Baker CJ, Long SS, McMillan JA, American Academy of Pediatrics (2007). Chlamydia trachomatis. Red Book.

[10] Carballal G, Mahony JB, Videla C, Cerqueiro C, Chernesky M (1992). Chlamydial antibodies in children with lower respiratory disease. Pediatr Infect Dis J.

[11] León A, Ceruti E, Díaz A, Pinto R, Farías P (1990). Etiología de las infecciones respiratorias agudas bajas en lactantes hospitalizados. Rev Chil Pediatr.

[12] Núñez RM, Duque J, Henríquez CW, Villegas AL, Niño JU, De Gonzalez B (1988). Viral and chlamydial etiology of acute infections of the lower respiratory tract in Colombian children. Pediatr Infect Dis J.

[13] Galán G, Martínez O, Cardoso D, Arango M, Castañeda E (1993). Chlamydia trachomatis como agente de neumonía en el Hospital de la Misericordia. Actual Pediatr.

[14] Dennis R (1989). Cómo estimar el tamaño de la muestra en investigaciones con humanos. Acta Med Colomb.

[15] Franco G (2005). Neumonitis viral un problema semestral.

[16] Furuya ME, Solórzano SF, Rendón ME, Zúñiga VG, Guerra IF, Aranda A (1994). Neumonitis por Chlamydia trachomatis en lactantes. Bol Med Hosp Infant Méx.

[17] Callahan CW (2005). Pneumonia and bacterial pulmonary infections. In:Panitch HB (ed.). Pediatric Pulmonology the requisites in pediatrics.. Philadelphia: Elsevier Mosby.

[18] Sant Anna  CC, C D Elia , Benguigui Y, López FJ, Schmunis G, Yunes J (1997). (eds). Infecciones respiratorias en niños.. Washington: OPS, OMS..

[19] Boyer KM, Feigin RD, Cherry JD, Demmler  GJ, Kaplan  SL (2004). Textbook of Pediatric Infectious Diseases. 5th ed. Philadelphia: Saunders..

[20] Korppi M (2003). Community-Acquired Pneumonia in children.. Paediatr Drugs..

[21] Rodríguez-Pastor SO, García FJ, Milano G (2001). Eritromicina y estenosis hipertrófica del píloro. An Esp Pediatr.

[22] Centers for Disease Control and Prevention (1999). Hypertrophic Pyloric stenosis in infants following pertussis prophylaxis with erythromycin, Knoxville, Tennessee. MMWR.

